# Global research hotpots on child weight management: visualized analysis based on CiteSpace

**DOI:** 10.3389/fpubh.2025.1665819

**Published:** 2025-11-27

**Authors:** Siyi Yang, Jing Wang, Hua Xu

**Affiliations:** Huzhou Central Hospital, Affiliated Central Hospital of Huzhou University, Huzhou, China

**Keywords:** children, weight management, CiteSpace, visualization analysis, bibliometrics

## Abstract

**Objective:**

This research aims to investigate the current research landscape, key focus areas, and emerging trends within the field of.

**Methods:**

We conducted searches on the Wanfang Data Knowledge Service Platform, China National Knowledge Infrastructure (CNKI), Web of Science (WOS) and PubMed databases. Bibliometric analysis was performed using CiteSpace 6.2.R3 software. Analytical methods included annual publication volume, co-authorship network analysis between countries, keywords co-occurrence analysis, and cluster analysis.

**Results:**

From January 1, 2022, to June 1, 2025, a total of 281 Chinese-language publications and 168 English-language publications were retrieved. China exhibited the highest publication output, followed by the largest number of publications in the world. China exhibited the highest publication output, followed by the United States and Italy. in current research, both domestically and internationally, centers on holistic lifestyle restructuring. This involves replacing unhealthy lifestyle patterns with structured health behavior interventions. This involves replacing unhealthy lifestyle patterns with structured health behavior interventions, such as personalized exercise prescriptions and precision nutrition plans. Cluster analysis identified 7 distinct research hotspots within the field. Citespace keyword timeline analysis shows that intervention studies in this field have consistently emphasized placing the participation of children and their families at the core.

**Conclusion:**

By conducting an in-depth exploration of the definition, characteristics, and influencing factors related to childhood weight management, alongside investigating intervention methods and measures, this by conducting an in-depth exploration of the definition characteristics, and influencing factors related to childhood weight management, alongside investigating intervention methods and measures, this research advocates for a paradigm shift. The field should transition from fragmented approaches toward standardized, multidisciplinary collaborative models for childhood weight management.

## 1 Introduction

In recent decades, the synergistic effects of global economic development and dietary habit transformations have contributed to a dramatic increase in childhood obesity prevalence worldwide, emerging as a critical public health challenge (1). The World Obesity Federation’s 2019 Childhood Obesity Atlas Report projects that approximately 254 million children and adolescents aged 5–19 years will be classified as obese globally by 2030 (2). The COVID-19 pandemic has further exacerbated this concerning trend through multiple pathways. Research indicates that pandemic control measures have led to significant behavioral changes, including increased food consumption with a shift toward less healthy options (e.g., processed meats and sugar-sweetened beverages), coupled with marked reductions in physical activity levels among children (3, 4). Notably, screen time among children has increased by 67% during this period, with the most pronounced increase (1.4 h per day) observed in children aged 6–10 years (5). Epidemiological evidence suggests that each additional daily hour of screen exposure may elevate obesity risk by approximately 2% (6).

Currently, there is a scarcity of systematic review studies on children’s weight management in China, with existing literature often lacking objective and quantitative analysis. CiteSpace, a widely recognized bibliometric analysis tool (7), has been extensively employed in review articles both domestically and internationally, demonstrating high reliability. Therefore, there is a pressing need for a more intuitive and bibliometrics-driven approach to systematically summarize research in this field, aiming to clarify the current research landscape, key focus areas, and emerging trends in children’s weight management globally. Such an analysis will provide valuable insights to inform the development of weight management interventions and guide clinical practice in China.

## 2 Data sources and research methods

### 2.1 Literature retrieval

#### 2.1.1 Search strategy

We conducted comprehensive literature searches in the Wanfang Database, China National Knowledge Infrastructure (CNKI), Web of Science (WOS), and PubMed databases to identify published research related to children’s weight management from January 1, 2022, to June 1, 2025. The Chinese search strategy employed was: (SU = ‘children’ + ‘obese children’ + ‘overweight children’) AND (SU = ‘weight’ OR ‘weight gain’ + ‘weight increase’ + ‘weight change’) AND (SU = ‘management’ + ‘intervention’ + ‘control’) AND (SU = ‘exercise’ OR ‘diet’). The English search strategy was: [TS = (“overweight children” OR “obese children”)] AND TS = (weight OR “weight gain” OR “body weight”) AND TS = (exercise* OR movement OR athletic OR sport* OR “physical activit*”) AND TS = (assessment OR management OR intervention OR control OR monitoring OR maintain). We limited the search to articles and review papers published in English.

#### 2.1.2 Inclusion criteria

(1) The literature is publicly published; (2) Patients are ≥18 years old; (3) Weight is one of the outcome indicators.

#### 2.1.3 Exclusion criteria

(1) The literature lacks the fields required for analysis; (2) The literature is a conference paper, meta-analysis, news article, patent, or scientific achievement; (3) The literature is a duplicate publication.

During the literature screening process, data were entered by two individuals. In cases of uncertainty, a third person made the determination, after which corrections were made.

### 2.2 Research methodology

CiteSpace, a bibliometric analysis tool developed by Dr. Chaomei Chen at Drexel University (USA) in collaboration with the WISE Laboratory of Dalian University of Science and Technology (DUST), enables the identification of research patterns through domain-specific literature analysis. In this study, CiteSpace 6.2.R3 was employed to process and visualize the retrieved literature. First, country and author collaboration networks were analyzed to identify core researchers and collaborative teams in the field. Subsequently, keyword co-occurrence analysis was conducted to detect high-frequency and high-centrality terms, generating a keyword network map. Keyword clustering was then applied to form thematic clusters (8), complemented by keyword burst detection to trace shifts in research perspectives and priorities. Through this multidimensional analytical framework, the study quantitatively and visually elucidates research hotspots and evolutionary trends in children’s weight management (9), thereby establishing a robust foundation for comprehensively mapping the current research landscape.

### 2.3 Procedure

**Figure 1:**
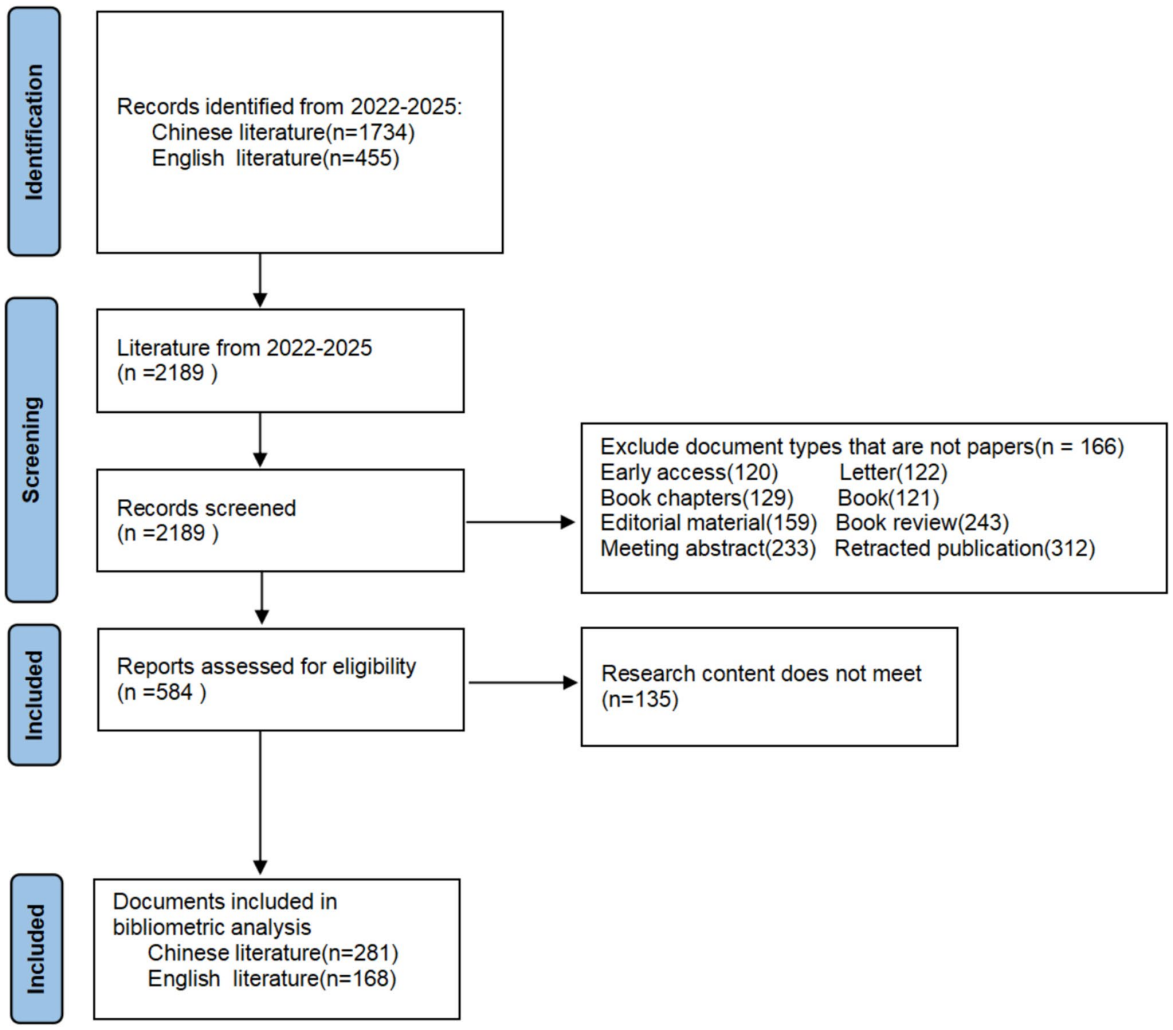
A PRISMA flowchart for bibliometric analysis and scoping of child weight management.

This study was conducted in four stages, as shown in the flowchart, ranging from data collection to visual presentation. Initially, a total of 2,189 papers related to “childhood weight management” were extracted from the Chinese and English core datasets, respectively. After screening, a total of 449 papers were ultimately included for analysis. Parameters were determined as follows: time slide = 2 years, top *N* = 50, clipping = PathFinder, and clustering algorithm = LLR (Figure 1).

## 3 Data results

### 3.1 Number of publications

**Figure 2:**
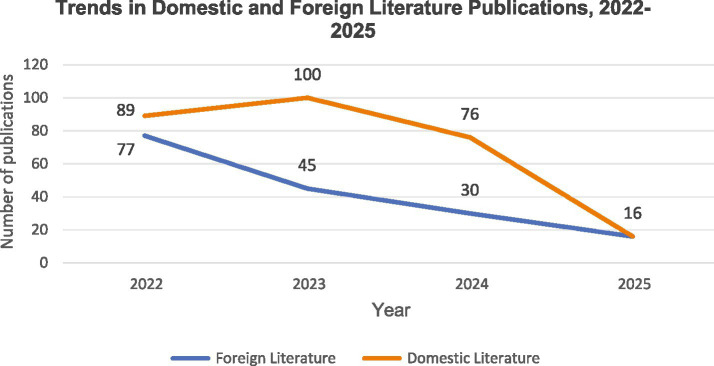
Annual trend chart of the number of articles published in domestic and foreign literature.

From January 1, 2022 to June 1, 2025, a total of 449 relevant publications were identified, comprising 281 Chinese-language articles and 168 English-language articles. As illustrated in Figure 2, domestic publications demonstrated a gradual increase starting in 2022, peaked in 2023, and subsequently entered a declining phase. Conversely, international publications exhibited a consistent year-on-year decrease since 2022.

### 3.2 Analysis of national cooperation mapping

**Figure 3:**
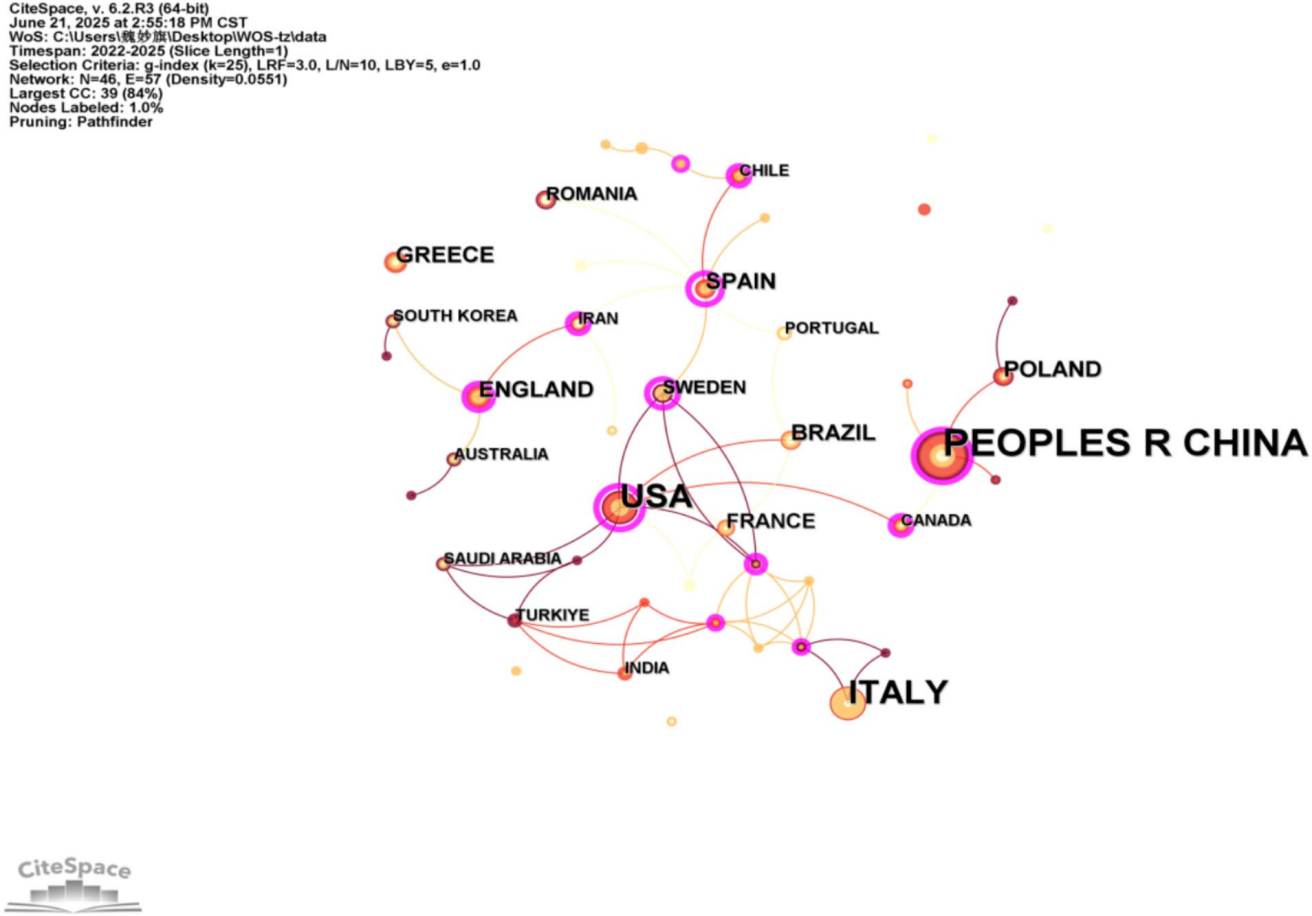
Knowledge map of country (region) distribution.

Using CiteSpace, we analyzed national publication volumes by selecting “Country” in the Node Types panel with a 1-year time interval. The resulting collaboration network (Figure 3) comprises 74 nodes and 101 links. In this visualization: the size of the nodes in the graph indicates the number of articles issued by the country (region); the color of the nodes indicates the cluster to which the country (region) belongs; the lines between the nodes indicate the existence of cooperative relationships, and the thicker the lines indicate the closer the relationship (10). As shown in Table 1, China contributed the highest number of publications, followed by the United States and Italy. In the international collaboration network, the China-Poland axis and the United States-Sweden axis both demonstrated close collaborative relationships, reflecting to some extent the synergistic advantages of these countries in terms of resource allocation and policy support. The development trends of these two collaborative axes illustrate the diversity of international cooperation models across different regional contexts. However, collaborations among most countries remain weak, indicating a need for gradually enhanced communication and deepened cooperation in the future.

**Table 1:** Top 10 countries in terms of frequency.

No.	Frequency	Centrality	Year	Countries
1	30	0.28	2022	PEOPLES R CHINA
2	18	0.63	2022	The Government of the United States of America (USA)
3	18	0	2022	ITALY
4	6	0.28	2023	ENGLAND
5	6	0.82	2022	SPAIN
6	6	0	2022	GREECE
7	6	0.07	2022	POLAND
8	6	0.05	2022	BRAZIL
9	5	0	2022	FRANCE
10	4	0	2022	ROMANIA

### 3.3 Keyword co-occurrence analysis

**Figure 4:**
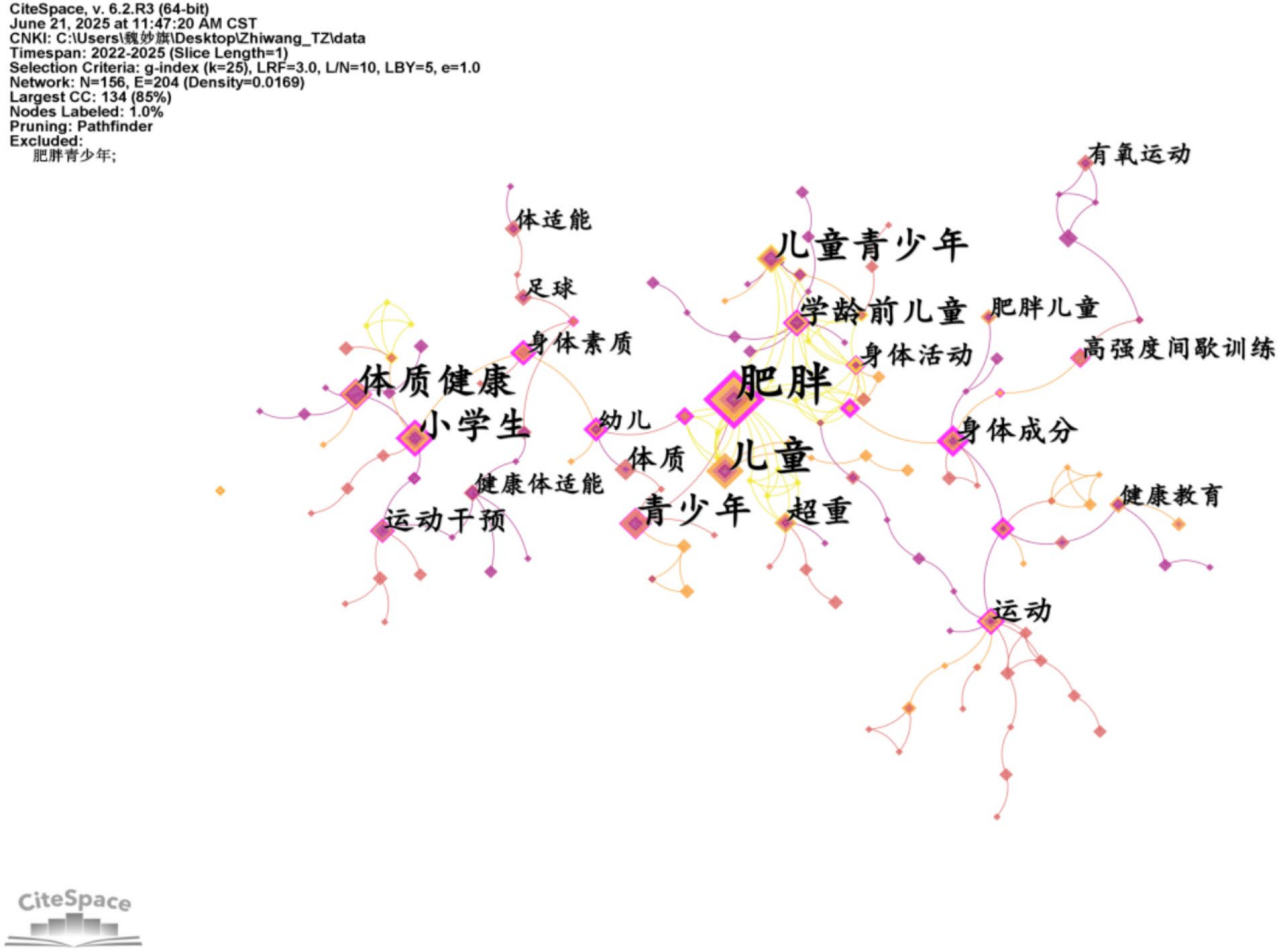
Keyword co-occurrence in Chinese scientific research.

**Figure 5:**
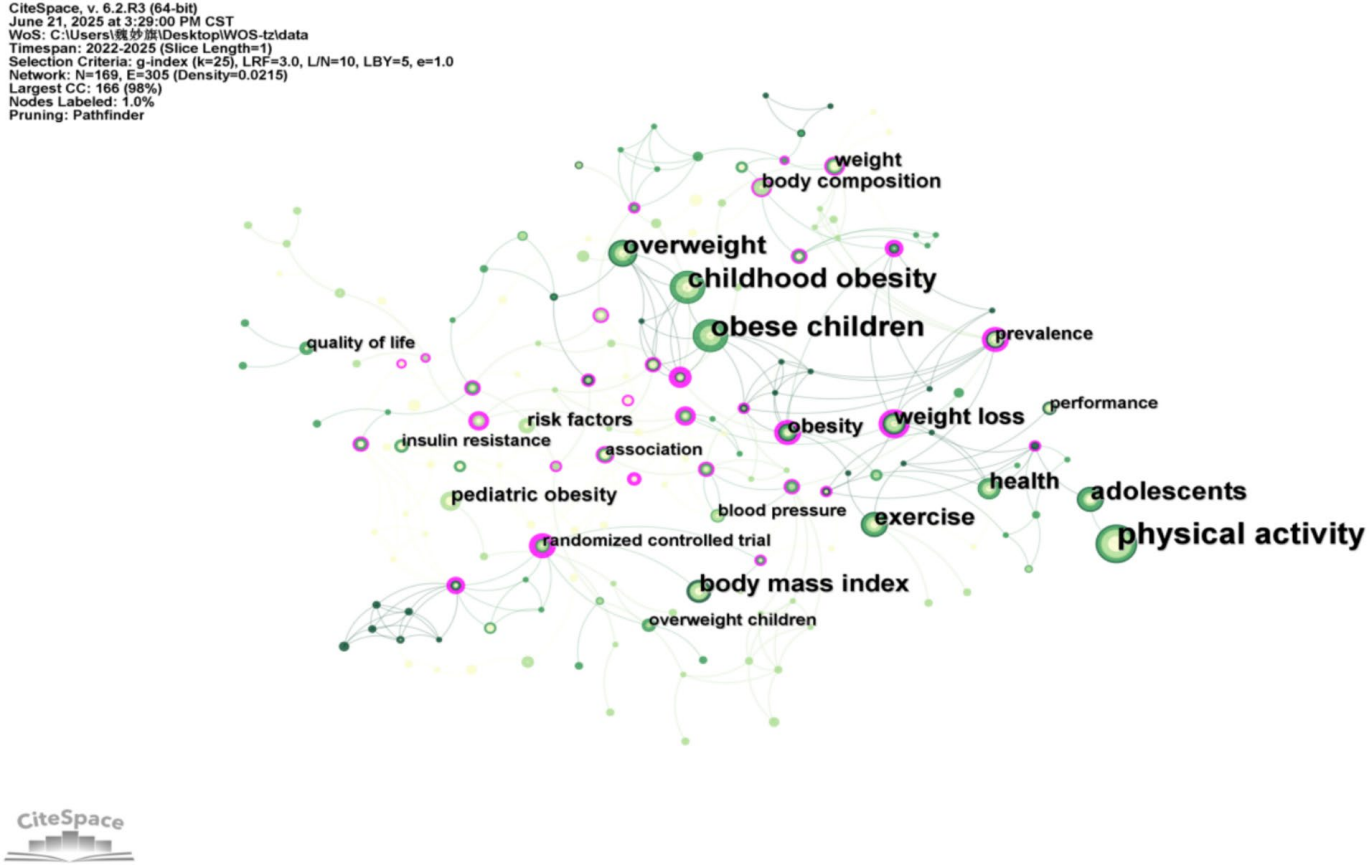
Keyword co-occurrence in international scientific research.

Keyword co-occurrence analysis identifies high-frequency terms and their interrelationships by quantifying the co-occurrence frequency of keywords within literature during a specified research period, thereby revealing core research foci. Based on the concepts of citation coupling and co-citation in bibliometrics (11), the intrinsic connections between keywords appearing in the same literature are analyzed to reveal the research themes or directions in the field. The higher frequency of co-occurring words in the literature represents the closer relationship between them. Table 2 lists the top 10 foreign keywords in this field in terms of frequency, which are physical activity (117 times), childhood obesity (102 times), body mass index (41 times), weight loss (29 times), and body composition (29 times), pediatric obesity (25 times), insulin resistance (24 times), metabolic syndrome (19 times), lifestyle intervention (17 times), and physical fitness (16 times). Next, the keywords will be analyzed for co-occurrence, and the keyword co-occurrence network will be drawn to explore the strength of the connection between the keywords, which is shown in Figures 4, 5. Figures 4, 5 conduct co-occurrence analysis of the keywords, mapping keyword co-occurrence networks to explore the strength of connections between keywords. The data presented in Figures 3, 4 reveal that the main high-frequency keywords in the field of "obesity" not only include the core term "children," but also other important keywords such as "physical activity," "exercise," and "physical health." The keyword co-occurrence network demonstrates strong interconnectivity, indicating that research on childhood weight management has accumulated substantial findings over the years and has established itself as a significant area of study (Table 3).

**Table 2:** The top 10 keywords in the frequency of domestic literature.

No.	Frequency	Centrality	Year	Keywords
1	45	0.88	2022	Obesity
2	29	0.09	2022	Children
3	19	0.05	2022	Children adolescents
4	19	0.13	2022	Physical fitness
5	18	0.11	2022	Adolescents
6	17	0.43	2022	Elementary school students
7	12	0.23	2022	Pre-school children
8	12	0.09	2022	Overweight
9	9	0.02	2022	Physical fitness
10	9	0.67	2022	Body composition

**Table 3:** The top 10 keywords in the frequency of foreign literature.

No.	Frequency	Centrality	Year	Keywords
1	55	0	2022	physical activity
2	41	0.08	2022	obese children
3	36	0.08	2022	childhood obesity
4	27	0.02	2022	adolescents
5	27	0.07	2022	overweight
6	22	0.02	2022	body mass index
7	0.06	0.06	2022	Exercise
8	18	0.07	2022	health
9	17	0.3	2022	Weight loss
10	13	0.24	2022	obesity

### 3.4 Keyword cluster analysis

**Figure 6:**
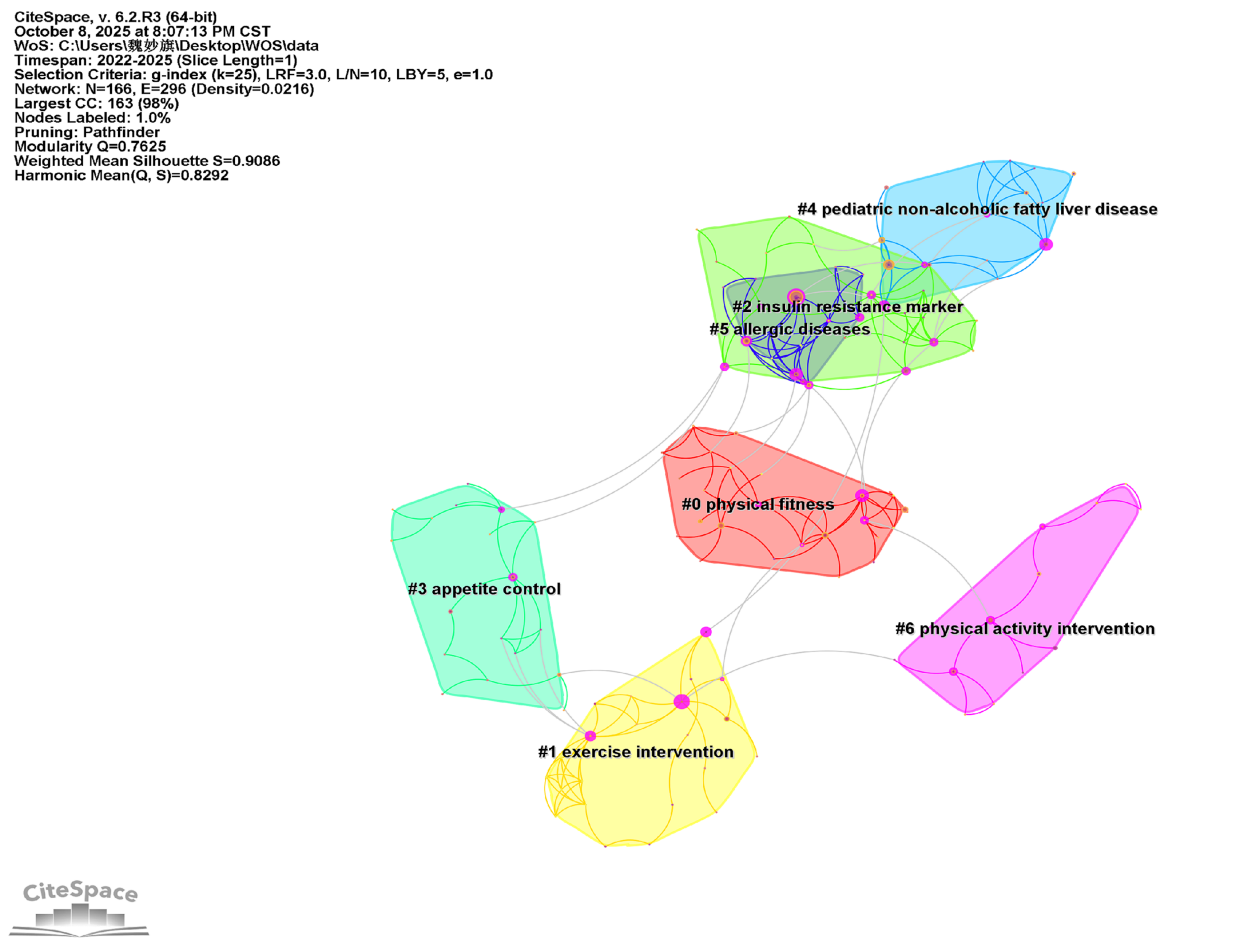
Clustering map of foreign children’s weight management keywords.

Keyword clustering analysis builds upon co-occurrence networks by employing algorithms (e.g., Log-Likelihood Ratio [LLR], Local Outlier Factor [LOF]) to synthesize keywords into numerically labeled clusters. Cluster IDs (e.g., #0, #1) follow size-based ordering, where smaller numbers indicate clusters containing more keywords. Each cluster label is determined by the keyword with the highest LLR value within that cluster, representing its core research theme. These clusters delineate the domain's knowledge structure and its evolution. In the visualization (Figure 6), distinct colors represent individual clusters, with keywords within each color range belonging to the corresponding thematic group (11). Based on keyword co-occurrence, this study conducted a clustering analysis of keywords and identified a total of 7 clusters, represented by irregular squares, namely: "physical fitness" (#0), "exercise intervention" (#1), "insulin resistance marker" (#2), "pediatric non-alcoholic fatty liver disease" (#3), "pediatric non-alcoholic fatty liver disease" (#4), "allergic diseases" (#5), and "physical activity intervention" (#6). As shown in Figure 5, the keyword co-occurrence map of research on childhood weight management generated 7 clusters. A modularity Q value of 0.7625 (>0.5) and a mean silhouette S value of 0.9086 (>0.7) indicate that the structure of the cluster analysis is significant. Furthermore, the figure highlights substantial overlap among clusters #2, #4, and #5, suggesting the presence of many shared keywords across these three clusters. "Allergic diseases" serves as an important bridge connecting cluster #2 "insulin resistance marker" and cluster #4 "pediatric non-alcoholic fatty liver disease". Cluster #2 has the smallest area, indicating it is composed of relatively few internal keywords.

### 3.5 Time line mapping analysis

**Figure 7:**
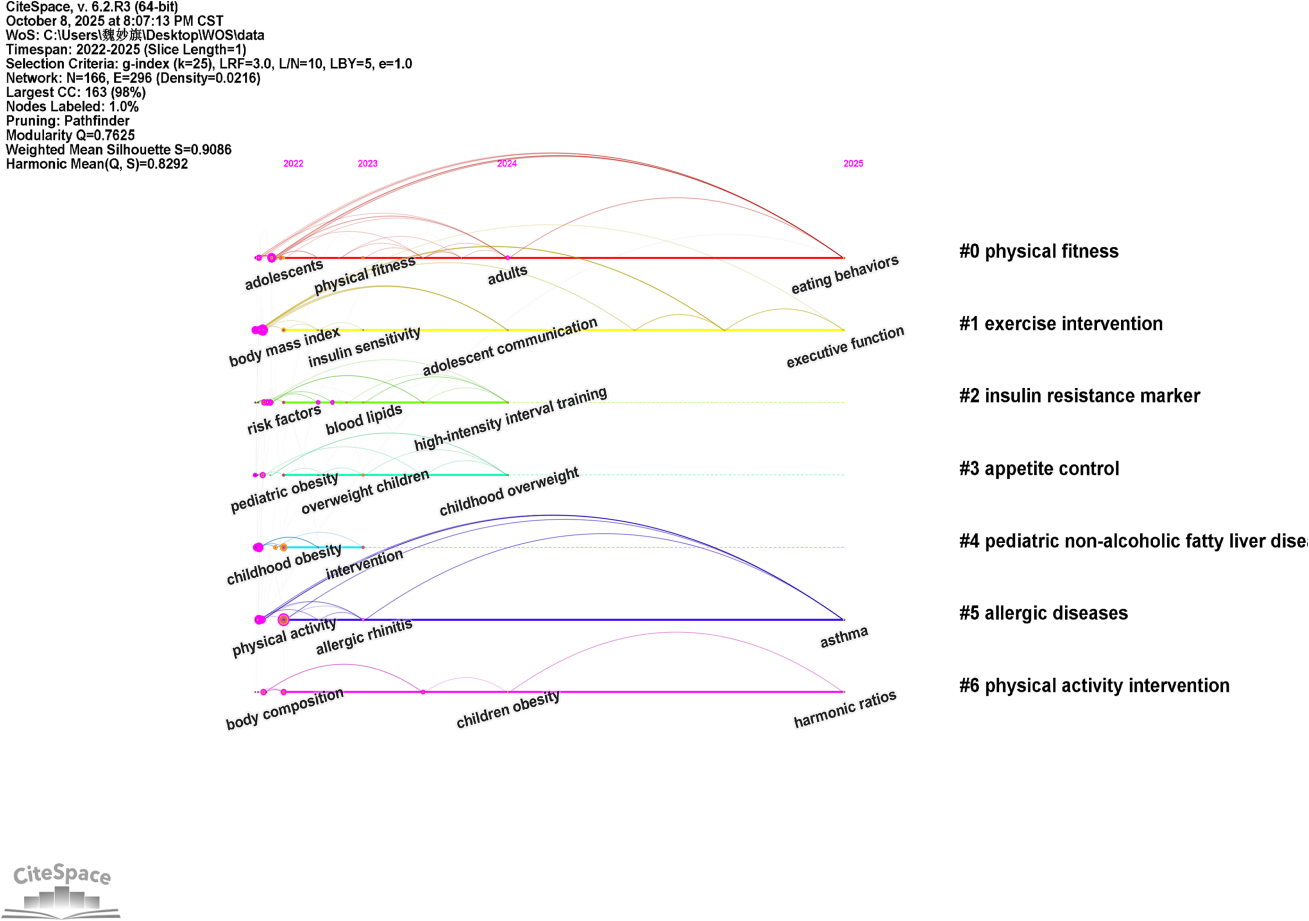
Timeline map of keywords for children’s weight management abroad.

In the timeline visualization (Figure 7), the abscissa represents the 2022–2025 timeline, progressing chronologically from left (early) to right (recent). The color gradient transitions from cool tones (blue/green) to warm tones (yellow/red) corresponding to temporal evolution. Annual vertical intervals demarcate research topic density fluctuations. The ordinate displays clustered research themes (e.g., #0, #1), with cluster labels positioned at the right margin (e.g., “mindfulness-based intervention,” “exercise intervention”). Node coloration indicates initial appearance time (cool: early; warm: recent). Nodes with purple outer rings denote high betweenness centrality (≥0.1), signifying pivotal knowledge bridges or disciplinary turning points. Inter-node connections represent co-occurrence/cocitation relationships, where line thickness correlates with association strength.

## 4 Discussion

### 4.1 Epidemiology of obesity and the urgency of weight management

Global obesity rates continue to escalate, demonstrating a trend toward earlier onset and stronger associations with chronic diseases. According to WHO data, the worldwide obese population has nearly tripled since 1975 (12). In China, approximately 20% of school-age children were overweight or obese in 2018, with projections indicating this prevalence may reach 31.8% by 2030 (13). Obesity-related medical expenditures now exceed 20% of total national healthcare costs, underscoring a critical public health burden. This crisis has elevated weight management from individual responsibility to national strategy. In 2024, China launched a three-year “National Weight Management Initiative,” mobilizing 16 governmental departments to create a supportive ecosystem for weight control. Evidence confirms that multisectoral collaboration enables sustainable obesity prevention through comprehensive, integrated approaches (14). Successful weight management research has moved from single calorie balancing to an era of multimodal precision interventions. International experience shows that effective management requires a four-dimensional linkage of policy and legislation, technological innovation, medical integration, and public education. Through the “Year of Weight Management,” China is exploring the prevention and control paths with local characteristics, but there is an urgent need to break through the bottlenecks of long-term effect maintenance and health economics evaluation. Future research should focus on biomarker mining, promotion of digital therapies, and cross-sectoral collaboration mechanisms, in order to realize the leap from “weight loss” to “whole-life health.”

### 4.2 Impact of overweight or obesity on children’s physical health

Overweight and obesity result from a chronic imbalance between energy intake and expenditure, which is characterized by an increased intake of energy-dense foods (e.g., fried foods, sugar-sweetened beverages) and physical inactivity resulting from a sedentary lifestyle. These dietary and inactivity patterns are strongly associated with environmental and social factors that promote the adoption of unhealthy behaviors, such as the intake of foods rich in sugars and fats or increased sedentary activity (15, 16). Childhood obesity has been associated with early markers of cardiovascular disease, insulin-resistant sleep disorders, increased risk of fractures, irregular menstruation in adolescent girls, and negative psychological effects (17, 18). In addition, obese children and adolescents are more likely to become obese adults and experience disability and premature death than their normal weight peers (19, 20). In the respiratory system, obese children have physiological limitations on chest wall expansion and diaphragmatic contraction due to excess abdominal adipose tissue, decreased chest compliance, and decreased lung compliance due to increased pulmonary blood flow and peripheral airway closure (21). Obesity decreases lung volume, increases airway resistance and affects lung capacity (22). Childhood obesity is an independent risk factor for obstructive sleep apnea (OSA), and the two conditions form a vicious cycle. Research hotspots focus on how intermittent hypoxia caused by OSA exacerbates insulin resistance and cardiovascular damage. The keyword timeline map analysis in Figure 6 shows that pediatric non-alcoholic fatty liver disease has become the leading cause of chronic liver disease in children. Related research hotspots concentrate on non-invasive diagnostic biomarkers (e.g., CK-18), the role of the gut-liver axis in pathogenesis, and the progression mechanisms from simple steatosis to steatohepatitis (23).

### 4.3 Effects of overweight or obesity on mental health aspects of children

Increasingly, boys and girls are experiencing negative emotions as a result of weight bullying Compared to their healthy weight peers, overweight or obese children are more likely to exhibit psychopathological manifestations, behavioral problems, and mood disorders (24), and they release negative emotions by avoiding physical education classes, consuming more food, and binge eating. Obese and overweight children are more likely to experience difficulties in peer relationships, and several studies have shown (25, 26) that such children have a higher probability of experiencing weight-related bullying than normal-weight children, and that such bullying is not moderated by gender, race, or socioeconomic background. At the same time, they are more likely to experience peer rejection and social isolation. Such rejection further exacerbates loneliness and internalized emotional problems (e.g., depression, anxiety), and obese children had significantly higher scores of low self-esteem than normal-weight children (*p* < 0.05), which was positively correlated with obesity (24). Obese children face weight bias from multiple environments, including from parents, obesity researchers, clinical settings (healthcare organizations), and schools. Parents not only show implicit bias against childhood obesity, but also implicit and explicit bias against obese children (27). This compromises their quality of life and promotes unhealthy behaviors that may exacerbate obesity, such as social isolation, decreased physical activity, and avoidance of medical care (28). Unfortunately, weight bias is widespread and tolerated in society, further extending the reach of its negative harm.

### 4.4 Analysis of research hotspots

In CiteSpace, research hotspots refer to the core themes that are common to the literature with intrinsic relevance within a specific time period, which are usually presented through keyword co-occurrence mapping and keyword clustering mapping. Keywords, as a condensation of the core content of the literature, focus on the core topics of the research.

#### 4.4.1 Exercise interventions in children’s weight management

Nemet D et al. (29) showed that a weight management program through a multidisciplinary association contributed to a decrease in BMI and BMI percentile in obese children and improved children’s health. The data showed that water aerobic intervention not only reduced the weight of obese children but also improved their lung function. Wang C et al. (30) showed that physical activity was effective in decreasing body weight, heart rate (HR), systolic blood pressure (SBP), and diastolic blood pressure (DBP), and increasing - vo2max (maximal oxygen uptake) in obese children and adolescents. Brown’s study found that that children’s physical activity levels can be increased through family involvement (31). In addition, Pamungkas studied home-based interventions for the treatment and prevention of childhood obesity, where home management could increase the frequency of physical activity and reduce sedentary time (32). Aerobic training, inverse group training, or combined exercise training were mainly used in home exercise studies, and both types of exercise were effective in reducing total body fat in children (33). The main objectives of home exercise therapy are to reduce total and visceral fat content in children and parents, increase lean body mass, accelerate the consumption of excess energy in the body, maintain energy balance in the body, increase resting metabolism levels, increase lipid metabolism levels, and reduce the incidence of metabolic and cardiovascular complications (34).

#### 4.4.2 Video games in weight management in children

In recent years, active video games have been proposed for obesity prevention and treatment as a potential tool to increase physical activity (35, 36). It requires physical activity to interact with on-screen images, reducing sedentary time and increasing metabolic equivalents through a technology based on body movement tracking (37). A study by Irandoust K et al. (38) demonstrated that the use of Xbox Kinect somatic gaming (39) as an intervention was effective in capitalizing on children’s interest in video games and significantly increased their participation in motivation for sports and exercise. This interest-driven intervention strategy not only enhanced exercise compliance, but also observed a significant improvement in first-second forceful expiratory volume of breath (FEV1) in the subject children, which in turn facilitated the achievement of weight control goals.

In the context of the information society, video games are increasingly recognized as a potential intervention tool for weight management. During the COVID-19 lockdowns, electronic sensor-based motion games were widely used to increase physical activity among children, effectively enhancing their daily activity levels during periods of home confinement (40).

## 5 Conclusion

This study reveals that childhood weight management research has evolved into a multidisciplinary field with clearly defined thematic clusters. Current research focuses on refining intervention strategies through structured physical activity programs, nutritional guidance, and family-centered behavioral modifications. The bibliometric analysis identifies seven major research clusters, with emerging emphasis on metabolic markers and personalized intervention approaches. Future research directions should prioritize: (1) Expanding the scope to include comorbidity profiles associated with childhood obesity; (2) Developing standardized biomarkers and objective assessment tools for early risk identification; (3) Accelerating the translation of evidence-based interventions into clinical practice through innovative delivery models; (4) Elucidating the physiological mechanisms linking childhood obesity to long-term health consequences. Establishing multidisciplinary collaborations, integrating advanced assessment technologies, strengthening mechanistic investigations, and implementing tailored intervention strategies represent critical steps toward advancing this field. The continuous evolution from fragmented approaches to integrated care models will ultimately enhance the efficacy of childhood weight management initiatives.

### 5.1 Research significance

The findings reveal that childhood weight management has formed a research architecture centered on physical activity interventions, metabolic markers, and specific disease clusters. Current studies demonstrate a transition from single-faceted behavioral interventions toward multidisciplinary coordinated care, with family involvement, personalized protocols, and long-term outcome evaluation emerging as critical research priorities.

(1) Deepening mechanistic investigations to elucidate the pathological relationships between obesity and childhood comorbidities such as metabolic abnormalities and non-alcoholic fatty liver disease.
(2) Developing integrated early-warning systems incorporating biomarkers, behavioral assessments, and environmental factors.
(3) Promoting innovative integrated intervention models operating within school-family-community settings.
(4) Enhancing comparative studies between Eastern and Western populations to establish localized guidelines tailored to the physiological characteristics of Chinese children.

We recommend establishing cross-disciplinary collaboration platforms, standardizing evaluation criteria, and strengthening the integration of evidence-based practice with real-world research to systematically advance childhood weight management from fragmented interventions toward a structured and precision-oriented prevention and control system.

### 5.2 Limitations

The data collection process focused solely on core Chinese and international databases. While these databases are widely utilized, they possess inherent limitations in terms of data coverage. Furthermore, a potential time lag exists between the publication of literature and its inclusion in these core databases. Newly published research may require a certain period before being indexed and searchable, which could introduce some bias into the findings. Therefore, future studies are recommended to incorporate a wider range of data sources to ensure comprehensiveness in data collection and minimize gaps in the research landscape.

## Data Availability

The original contributions presented in the study are included in the article/Supplementary material, further inquiries can be directed to the corresponding authors.
